# The Prognostic Value of lncRNA MCM3AP-AS1 on Clinical Outcomes in Various Cancers: A Meta- and Bioinformatics Analysis

**DOI:** 10.1155/2022/4466776

**Published:** 2022-06-24

**Authors:** Liangyin Fu, Guangming Zhang, Yongfeng Wang, Tingting Lu, Bin Liu, Yajun Jiao, Haizhong Ma, Shixun Ma, Kehu Yang, Hui Cai

**Affiliations:** ^1^The First Clinical Medical College of Gansu University of Chinese Medicine (Gansu Provincial Hospital), Lanzhou 730000, China; ^2^General Surgery Clinical Medical Center, Gansu Provincial Hospital, Lanzhou 730000, China; ^3^Key Laboratory of Molecular Diagnostics and Precision Medicine for Surgical Oncology in Gansu Province, Gansu Provincial Hospital, Lanzhou 730000, China; ^4^Gansu Provincial Hospital, Lanzhou 730000, China; ^5^Evidence-Based Medicine Center, School of Basic Medical Sciences, Lanzhou University, Lanzhou 730000, China; ^6^NHC Key Laboratory of Diagnosis and Therapy of Gastrointestinal Tumor, Gansu Provincial Hospital, Lanzhou 730000, China

## Abstract

**Background:**

MCM3AP antisense RNA 1 (MCM3AP-AS1) is a newly identified potential tumor biomarker. Nevertheless, the prognostic value of MCM3AP-AS1 in cancer has been inconsistent in the available studies. We performed this meta-analysis to identify the prognostic role of MCM3AP-AS1 in various cancers.

**Methods:**

We searched PubMed, Web of Science, EMBASE, and the Cochrane Library databases to screen relevant studies. Hazard ratios (HR) or odds ratios (OR) and corresponding 95% confidence intervals (CI) were used to evaluate the relationship between aberrant MCM3AP-AS1 expression and survival and clinicopathological features (CFS) of cancer patients. A meta-analysis was performed using STATA 12.0 software. Additionally, results were validated by an online database based on The Cancer Genome Atlas (TCGA). Subsequently, we analyzed the MCM3AP-AS1-related genes and molecular mechanisms based on the MEM database.

**Results:**

Our results showed that overexpression of MCM3AP-AS1 was related to poor overall survival (OS) (HR = 2.00, 95% CI, 1.52–2.64, *P* < 0.001) and relapse-free survival (RFS) (HR = 3.28, 95% CI 1.56–6.88, *P* = 0.002). In addition, MCM3AP-AS1 overexpression was associated with TNM stage, differentiation grade, and lymph node metastasis, but not significantly with age, gender, and tumor size. In addition, MCM3AP-AS1 overexpression was verified by the GEPIA online database to be associated with poorer survival. The further functional investigation suggested that MCM3AP-AS1 may be involved in several cancer-related pathways.

**Conclusions:**

The overexpression of MCM3AP-AS1 was related to poor survival and CFS. MCM3AP-AS1 may be considered a novel prognostic marker and therapeutic target in various cancers.

## 1. Introduction

Cancer threatens human health, is a leading cause of death, and is a major obstacle to increasing life expectancy in countries worldwide [1, 2]. While significant advances in cancer research have been made, the treatments developed and patient prognosis have not met expectations, necessitating a change in how cancer is researched and treated [3]. Numerous cancers can be prevented or effectively treated if diagnosed early [4]. The presence of tumor markers helps in the early detection of cancer [5]. Thus, looking into novel tumor markers, finding tumors earlier, and treating patients immediately and effectively can help to improve their prognosis.

Long noncoding RNA (lncRNA) is a noncoding transcript with a length larger than 200 nucleotides, which cannot encode proteins owing to open reading frame deficiency [6, 7]. Through continuous research, lncRNA has been identified to be engaged in transcriptional and posttranscriptional regulation by interacting with DNA, RNA, or proteins and regulates various physiological and pathological processes [8–10]. Aberrant expression of lncRNA acts as suppressor genes or oncogenes and is involved in tumorigenesis, progression, and metastasis [11]. Therefore, lncRNAs with distinctive expression and functional variety can be regarded a diagnostic and prognostic biomarker and may provide new therapeutic targets for the clinic [12, 13].

MCM3AP antisense RNA 1 (MCM3AP-AS1) is a novel lncRNA located on chromosome 21 at places 46,228,977-46,259,390. It is found that subcellular localization is chromatin and nucleoplasm [14]. Recent studies have found that MCM3AP-AS1 is aberrantly expressed in a variety of human cancers and usually predicts poor prognosis in several cancers, including breast cancer [15], colorectal cancer (CRC) [16], endometrioid carcinoma (EC) [17], hepatocellular carcinoma (HCC) [18], lung cancer (LC) [19], nasopharyngeal carcinoma (NPC) [20], oral squamous cell carcinoma (OSCC) [21], pancreatic cancer (PC) [22], prostate cancer (PCa) [23], and renal cell carcinoma (RCC) [24]. Meanwhile, abnormal expression of MCM3AP-AS1 is associated with clinicopathological features (CFS) of various cancers, such as tumor size, tumor stage, lymph node metastasis, and distant metastasis. Moreover, the expression of MCM3AP-AS1 influences the development and progression of numerous cancers. MCM3AP-AS1 is highly expressed in breast cancer cells and promotes tumor growth by targeting centromere protein F (CENPF) [25]. MCM3AP-AS1 silencing inhibited the proliferation and migration of CRC cells [14]. In summary, MCM3AP-AS1 may be a novel tumor marker and therapeutic target. However, since most published research is limited by a low sample size, the prognostic value of expression of the lncRNA MCM3AP-AS1 remains unclear. Therefore, we conducted this meta-analysis to explore the relationship between lncRNA MCM3AP-AS1 expression and overall survival (OS), relapse-free survival (RFS), and CFS.

The fast development of bioinformatics provides a broad prospect for the research of disease diagnosis and therapeutic targets [26]. For example, Lee et al. found that *DLK2* acts as a potential prognostic biomarker for RCC based on bioinformatics analysis [27]. Zhou et al. suggested that patients with *CYB561* overexpression have reduced OS and increased risk of death, and *CYB561* may serve as a valid clinical prognostic biomarker for breast cancer [28]. Therefore, to further understand the prognostic potential of lncRNA MCM3AP-AS1 expression, we performed bioinformatics analysis to investigate the potential prognostic value of MCM3AP-AS1 in cancers. In addition, we explored the genes and pathways associated with MCM3AP-AS1. To better guide the clinical work, we intend to explore the potential of MCM3AP-AS1 as a novel tumor marker and therapeutic target.

## 2. Materials and Methods

### 2.1. Registration Subheadings

Our meta-analysis was registered on PROSPERO (ID: CRD42021293772).

### 2.2. Search Strategy

Two authors independently searched PubMed, Web of Science, EMBASE, and the Cochrane Library databases. Our search terms were used as follows: (“MCM3AP-AS1” OR “MCM3APAS” OR “MCM3AP-AS” OR “MCM3AP antisense RNA 1” OR “long noncoding RNA MCM3AP-AS1” OR “lncRNA MCM3AP-AS1” OR “long non-coding RNA MCM3AP-AS1”) AND (“tumor” or “cancer” or “carcinoma” or “neoplasm” or “sarcoma” or “melanoma” or “adenoma).

### 2.3. Inclusion and Exclusion Criteria

The inclusion criteria were as follows: (i) the expression level of MCM3AP-AS1 in tumor tissues was detected and divided into two groups of high and low expression; (ii) provides information on the association of MCM3AP-AS1 with survival or CFS; (iii) reported hazard ratio (HR) for OS and RFS or provided survival curves to allow calculation of HR; and (iv) all data were obtained from clinical samples. The exclusion criteria were as follows: (i) reviews, case reports, conference abstracts, letters, or animal studies; (ii) studies without survival or clinicopathological data; and (iii) data from the database.

### 2.4. Data Extraction and Quality Assessment

Two authors independently screened for inclusion in the study and extracted the required information and data [29]. When there was disagreement, a third author intervened to reach a consensus. Based on the inclusion and exclusion criteria, the following information was extracted: (i) name of first author and year of publication, (ii) country of publication, (iii) tumor type, (iv) sample size, (v) lncRNA MCM3AP-AS1 detection method, (vi) cut-off value, (vii) follow-up time, (viii) outcomes, and (ix) OS and RFS data. We evaluated the quality of the included studies according to the Newcastle-Ottawa Scale [30] (NOS), which used nine entries to assess studies, with one point for each entry satisfied and a total score between 0 and 9. Based on the scores obtained, they were classified as high quality (7-9), moderate quality (4-6), and low quality (0-3). All scoring was done independently by two authors.

### 2.5. Validation by Reviewing Public Data

Gene Expression Profiling Interactive Analysis (GEPIA) is based on The Cancer Genome Atlas (TCGA) and can be used to validate gene differential expression analysis in tumor/normal tissues [31]. Our meta-analysis used GEPIA to validate the association of MCM3AP-AS1 expression with OS and detect the distinction of MCM3AP-AS1 expression levels between normal and tumor tissues. Survival analysis was performed using the K-M method and log-rank test, and the figure of K-M curves displayed the HR and *P* value.

### 2.6. Predicting Target Genes and Building Signal Pathway Network

We acquired MCM3AP-AS1-related genes from the MEM database [32] (https://biit.cs.ut.ee/mem/index.cgi). Later, we performed gene ontology (GO) and the Kyoto Encyclopedia of Genes and Genomes (KEGG) enrichment analysis on the obtained genes by online databases (http://www.bioinformatics.com.cn). Furthermore, we constructed and visualized the MCM3AP-AS1-related signaling pathway network using Cytoscape software [33].

### 2.7. Statistical Analysis

We predicted the correlation between MCM3AP-AS1 expression and tumor patients' survival based on HR and 95% confidence interval (CI). Some of the included studies had precise survival data that could be utilized directly. For the remaining studies that only provided KM curves, we used Engauge Digitizer V.4.1 software to extract survival data and calculate HR and 95% CI [34]. Survival outcomes were expressed by log HR and standard error (SE), and clinicopathological parameters were expressed by odds ratio (OR) and 95% CI. Between-study heterogeneity was assessed using chi-squared tests and the *I*^2^ statistic. We used a fixed-effects model to analyze the results when *I*^2^ < 50% and the *P* value of *Q* test (*PQ*) ≥ 0.05. Otherwise, a random-effects model was used. If there was significant heterogeneity between studies, subgroup analysis was used to find the source of heterogeneity. Meta-analysis outcomes were shown using forest plots. Begg's funnel plot and Egger's regression test were used to evaluate publication bias. To assess the stability of the effect of independent studies on the results, we performed a sensitivity analysis on this. The study results were analyzed using STATA 12.0, and *P* < 0.05 was deemed statistically significant.

## 3. Results

### 3.1. Characteristics of Studies

**Figure 1:**
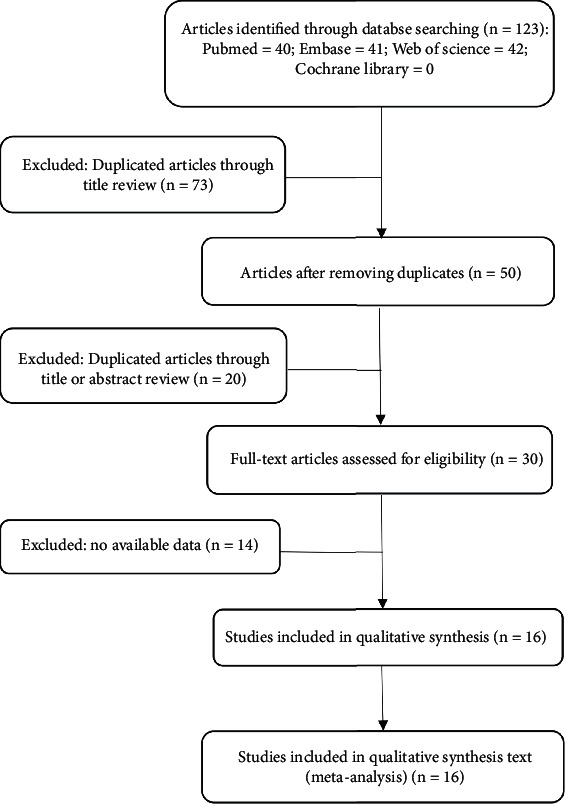
Flow diagram of literature screening for meta-analysis.

We retrieved a total of 123 articles from the four databases (PubMed, Web of Science, EMBASE, and the Cochrane Library), and 16 studies were finally included through screening.  Figure 1 (Figure 1) shows the process and results of screening the literature according to PRISMA criteria. All the included studies were published in 2019-2021 and were from China. Ultimately, the included studies included 12 types of cancer, such as cervical carcinoma (CC) [35], CRC [16, 36, 37], EC [17], HCC [38], LC [19, 39], lymphoma [40], NPC [20], OSCC [21], PC [22], papillary thyroid cancer (PTC) [41], PCa [23, 42], and RCC [24]. There was sufficient data for OS and RFS to be considered as survival outcomes, and Table 1 demonstrated the basic characteristics of these studies.

### 3.2. Association of MCM3AP-AS1 Expression Levels with OS and RFS

**Figure 2:**
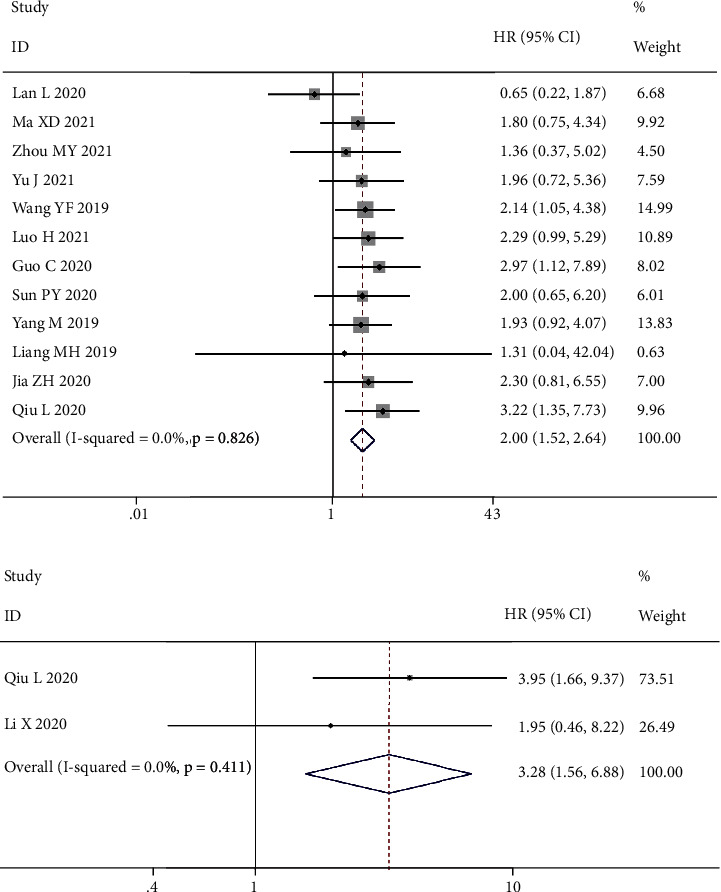
Forest plots for the association of MCM3AP-AS1 expression with overall survival (a) and relapse-free survival (b).

Figure 2(a) shows the relationship between MCM3AP-AS1 expression and OS. Twelve studies with 816 patients were included, and all the data were obtained from clinical samples. We used a fixed-effects model since these studies had no heterogeneity (*I*^2^ = 0.0%, *PQ* = 0.826). Meta-analysis results showed that tumor patients with high MCM3AP-AS1 expression had poor OS (HR = 2.00, 95% CI 1.52–2.64, *P* < 0.001) (Figure 2(a)). Therefore, MCM3AP-AS1 was an independent factor in the low survival of tumor patients. In addition, two studies were included for RFS analysis. The fixed-effect model was applied (*I*^2^ = 0%, *PQ* = 0.411). The results indicated that high MCM3AP-AS1 expression predicts poor RFS in patients (HR = 3.28, 95% CI 1.56–6.88, *P* = 0.002) (Figure 2(b)).

### 3.3. Subgroup Analysis of the Association between MCM3AP-AS1 Expression Level and OS

**Figure 3:**
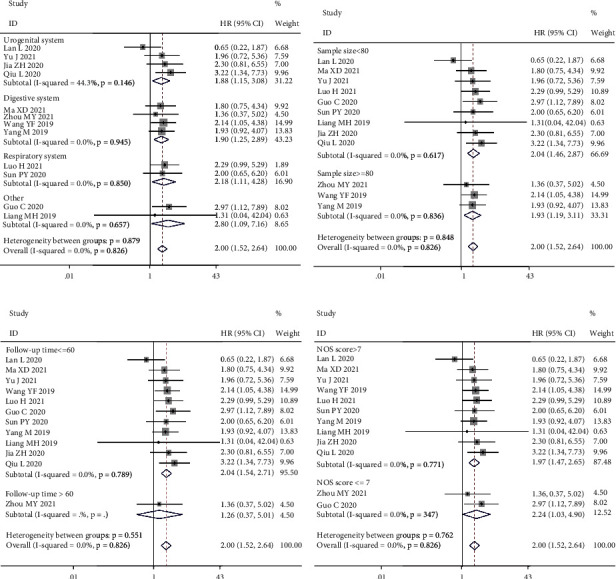
Forest plots for subgroup analysis of MCM3AP-AS1 expression with overall survival. (a) Subgroup analysis stratified by the system of cancers. (b) Subgroup analysis stratified by sample size. (c) Subgroup analysis stratified by follow-up time. (d) Subgroup analysis stratified by NOS score.

To further assess the relationship between MCM3AP-AS1 expression levels and OS, we performed a subgroup analysis based on the following factors: the system of cancers (digestive system, urogenital system, respiratory system, or other) (Figure 3(a)), sample size (≥80 < 80 tissues) (Figure 3(b)), follow-up time (>60 or ≤60 months) (Figure 3(c)), and article quality (NOS scores ≥ 7 or <7) (Figure 3(d)). The outcomes of the subgroup analysis did not change the predictive value of MCM3AP-AS1 for OS in cancer patients.

### 3.4. Association between MCM3AP-AS1 and Clinicopathological Features

**Figure 4:**
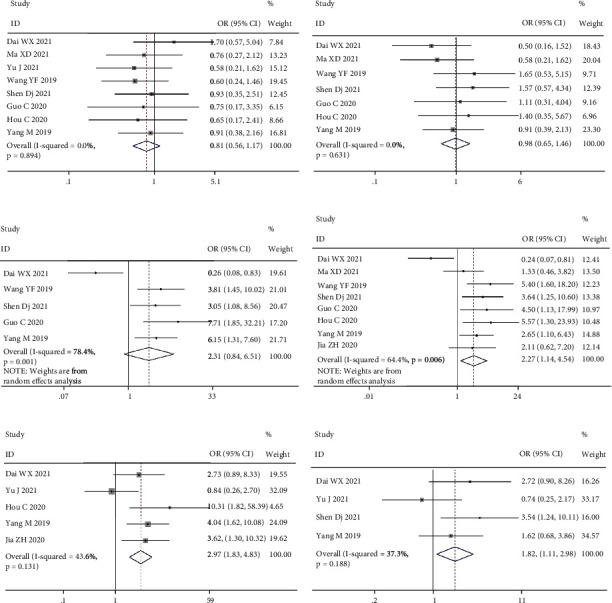
Forest plot for the association between MCM3AP-AS1 expression levels and age (a), gender (b), tumor size (c), TNM stage (d), lymph node metastasis (e), and differentiation grade (f).

The correlation between MCM3AP-AS1 expression level and CFS was investigated using OR and the 95% CI. As shown in the meta-analysis results in Figure 4 and Table 2, the overexpression of MCM3AP-AS1 was significantly related to TNM stage (OR = 2.28, 95% CI 1.14–4.54, *P* = 0.019, Figure 4(d)), differentiation grade (OR = 1.82, 95% CI 1.11–2.98, *P* = 0.018, Figure 4(f)), and lymph node metastasis (OR = 2.97, 95% CI 1.83-4.83, *P* < 0.001, Figure 4(e)). However, MCM3AP-AS1 expression was not significantly correlated with age (OR = 0.81, 95% CI 0.56–1.17, *P* = 0.263, Figure 4(a)), gender (OR = 0.98, 95% CI 0.65–1.46, *P* = 0.902, Figure 4(b)), and tumor size (OR = 2.34, 95% CI 0.84– 6.51, *P* = 0.105, Figure 4(c)).

### 3.5. Sensitivity Analysis and Publication Bias

**Figure 5:**
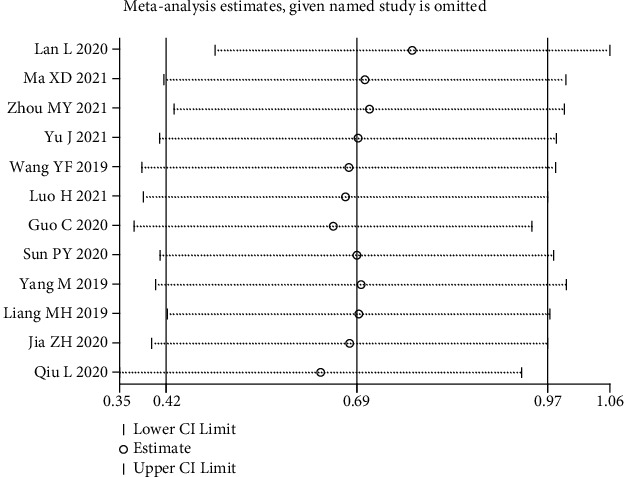
Sensitivity analysis of pooled HR for overall survival.

**Figure 6:**
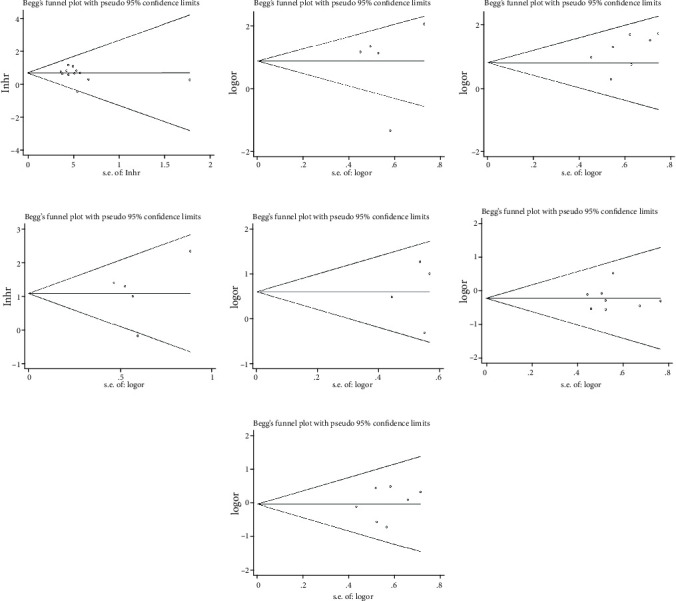
Begg' s publication bias plots: overall survival (a), tumor size (b), TNM stage (c), lymph node metastasis (d), differentiation grade (e), age (f), and gender (g).

To assess the effect of each independent study on the OS results, we performed a sensitivity analysis. After excluding each eligible study, the outcomes did not change significantly, thus substantiating the robustness of the meta-analysis results and the reliability of MCM3AP-AS1 expression on OS prediction (Figure 5). Begg's funnel plot and Egger's regression test were used to investigate possible publication bias. Our results revealed no obvious publication bias for OS (*P* > |*t*| = 0.382; Figure 6(a)), tumor size (*P* > |*t*| = 0.939; Figure 6(b)), TNM stage (*P* > |*t*| = 0.729; Figure 6(c)), lymph node metastasis (*P* > |*t*| = 0.750; Figure 6(d)), differentiation grade (*P* > |*t*| = 0.883; Figure 6(e)), age (*P* > |*t*| = 0.972; Figure 6(f)), and gender (*P* > |*t*| = 0.599; Figure 6(g)).

### 3.6. Validation of the Results in TCGA Dataset

**Figure 7:**
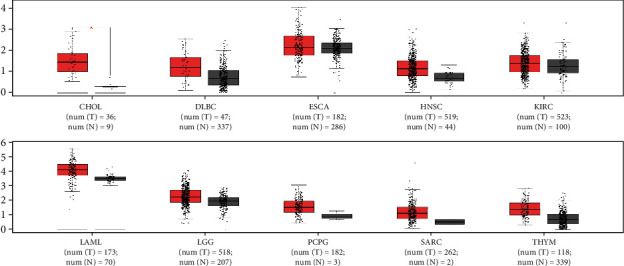
The expression levels of MCM3AP-AS1 in CHOL (cholangiocarcinoma), DLBC (lymphoid neoplasm diffuse large B-cell lymphoma), ESCA (esophageal carcinoma), HNSC (head and neck squamous cell carcinoma), KIRC (kidney renal clear cell carcinoma), LAML (acute myeloid leukemia), LGG (brain lower grade glioma), PCPG (pheochromocytoma and paraganglioma), SARC (sarcoma), and THYM (thymoma). The red box plots represent MCM3AP-AS1 expression in cancer tissues, and the grey box plots represent MCM3AP-AS1 expression in normal tissues.

To further verify our results, we investigated the expression levels of MCM3AP-AS1 in various cancers using TCGA dataset. The results showed that MCM3AP-AS1 expression was upregulated in a variety of cancers, including cholangiocarcinoma (CHOL), esophageal carcinoma (ESCA), lymphoid neoplasm diffuse large B-cell lymphoma (DLBC), kidney renal clear cell carcinoma (KIRC), head and neck squamous cell carcinoma (HNSC), brain lower grade glioma (LGG), acute myeloid leukemia (LAML), pheochromocytoma and paraganglioma (PCPG), sarcoma (SARC), and thymoma (THYM) (Figure 7).

**Figure 8:**
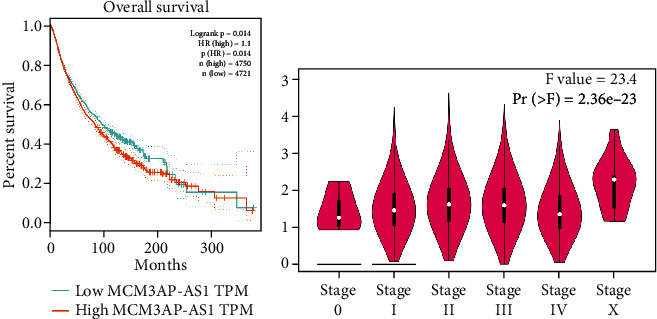
(a) Overall survival plot of MCM3AP-AS1 in the GEPIA cohort (*n* = 9471). (b) Violin plot showing MCM3AP-AS1 expression in different major clinical stages of pancancers in the GEPIA cohort.

Furthermore, by combining MCM3AP-AS1 expression data from all TCGA databases and OS data of human tumors, the GEPIA survival plots were used to divide 9471 patients into the MCM3AP-AS1 high-expression group and the MCM3AP-AS1 low-expression group. The results showed that upregulation of MCM3AP-AS1 expression predicted poorer OS, confirming the results of our meta-analysis (Figure 8(a)). Moreover, the violin plot showed that the expression level of MCM3AP-AS1 was significantly related to the clinical stages of human tumors (Figure 8(b)).

### 3.7. Analysis of MCM3AP-AS1-Related Genes

**Figure 9:**
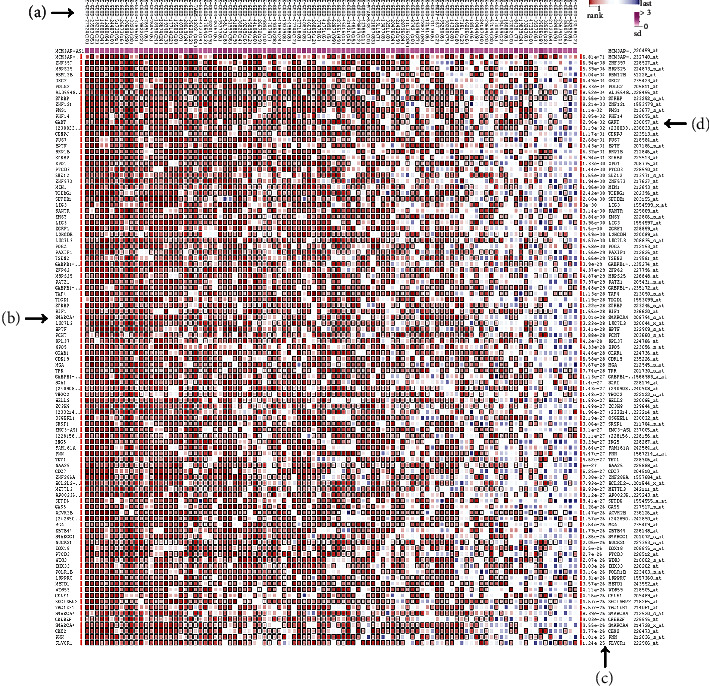
The top 100 predicted target genes of lncRNA MCM3AP-AS1 by using the MEM database: (a) one experimental dataset; (b) predicted target genes; (c) *P* values; (d) gene probes.

**Figure 10:**
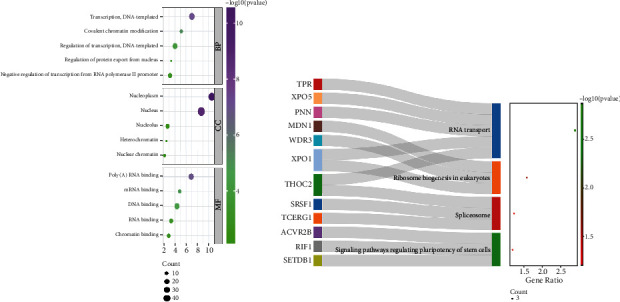
GO terms and the KEGG pathway. (a) GO enrichment of target genes in BP (biological process), CC (cellular component), and MF (molecular function) ontology. (b) The pathways related to the differentially expressed genes by the KEGG database analysis. GO: gene ontology analysis; KEGG: Kyoto Encyclopedia of Genes and Genomes.

**Figure 11:**
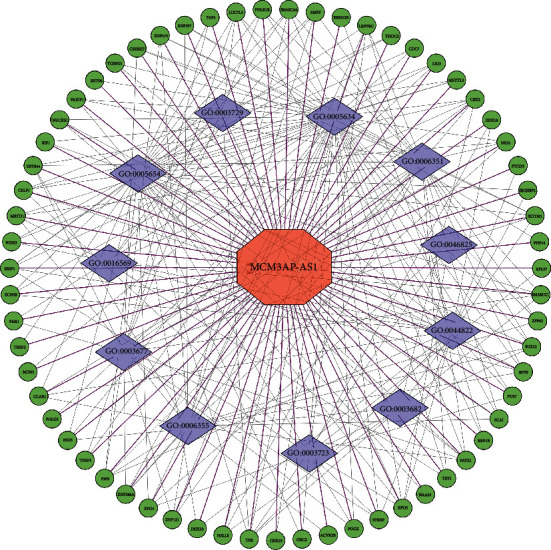
Differentially expressed gene interaction network analysis. Green nodes represent target genes, and purple nodes represent the related pathway. As indicated in red, MCM3AP-AS1 is localized at the center of the network.

We filtered the top 100 MCM3AP-AS1-related genes from the MEM database and found that *ZNF397*, *MRPS25*, and *RBM12B* were the top three predicted target genes, closely associated with MCM3AP-AS1 gene expression (Figure 9). Furthermore, we used GO and KEGG enrichment analysis to understand the potential molecular mechanisms of MCM3AP-AS1 in cancer (Figure 10; Table 3). Also, we used Cytoscape software to make a signaling pathway network of these MCM3AP-AS1-related genes that coexpressed with MCM3AP-AS1 (Figure 11).

## 4. Discussion

Cancer remains a major public health problem worldwide and is one of the leading causes of death in every country [43]. In the past two years, cancer incidence and mortality rates have increased further due to delays in the diagnosis and treatment of cancer due to the novel coronavirus [44]. However, early cancer detection and advances in treatment can improve patient survival rates [45]. It has been shown that many lncRNAs are abnormally expressed in diverse cancers. lncRNA can influence cancer development and progression by accelerating tumor cell proliferation, metastasis, and invasion [46]. Furthermore, because of their tissue specificity and stability, lncRNAs have the potential to be therapeutic targets as well as diagnostic or prognostic biomarkers [12]. Therefore, lncRNA is an important biomarker for cancer diagnosis and treatment, and it could be used as a possible therapeutic target to improve the prognosis of people with cancer.

As a study reported, several lncRNAs play an essential part in the tumor occurrence and development of different cancers [47]. For example, Fang et al. [48] found that lncRNA SLCO4A1-AS1 was highly upregulated in GC and accelerates growth and metastasis of GC. Furthermore, they conclude that SLCO4A1-AS1 is an important oncogenic lncRNA in GC, and SLCO4A1-AS1 is a potential novel therapeutic target for GC. A study by Bhan et al. suggest that lncRNA PVT1 accelerates breast cancer proliferation and metastasis as an oncogene and may be a potential therapeutic target for breast cancer. Therefore, it is crucial to identify new tumor markers associated with the prognosis of malignant tumors. lncRNA can be considered as molecular marker for tumors, and its expression can be used to predict tumor prognosis and patient prognosis, providing a new basis for cancer diagnosis and treatment [49].

In recent years, MCM3AP-AS1 is a novel lncRNA and it was found to be aberrantly expressed in multiple cancers, including CC [35], CRC [16, 36, 37], EC [17], HCC [38], LC [19, 39], lymphoma [40], NPC [20], OSCC [21], PC [22], PTC [41], PCa [23, 42], and RCC [24]. MCMAP-AS1 has the possibility of being a novel molecular marker and therapeutic target. Wang et al. revealed that lncRNA MCM3AP-AS1 was overexpressed in HCC and was related to poor prognosis, advanced tumor stage, high tumor grade, and large tumor size in HCC patients [38]. Ma et al. showed that lncRNA MCM3AP-AS1 was upregulated in CRC and that MCM3AP-AS1 overexpression was associated with poor survival of CRC patients [36]. Shen et al. found that MCM3AP-AS1 was overexpressed in non-small-cell lung cancer (NSCLC), and MCM3AP-AS1 may be a promising therapeutic target for NSCLC patients [39]. Yu et al. demonstrated that MCM3AP-AS1 was upregulated in EC and presented poor survival [17].

We integrated existing studies exploring the association between MCM3AP-AS1 and OS, RFS, and CFS of cancer patients and performed a meta-analysis to assess the potential of MCM3AP-AS1 as a therapeutic target and prognostic marker for cancer. The results showed that lncRNA MCM3AP-AS1 expression was upregulated in various cancers, but Lan et al. and Dai et al. found that MCM3AP-AS1 expression was downregulated. Furthermore, our findings revealed that cancer patients with overexpression of lncRNA MCM3AP-AS1 had poorer OS and RFS. Regarding CFS, we found that overexpression of MCM3AP-AS1 correlated with TNM stage, lymph node metastasis, and differentiation grade, independent of age, gender, and tumor size. Therefore, we suggested that overexpression of MCM3AP-AS1 was closely related to patients' poor prognosis and CFS; MCM3AP-AS1 can be used as a diagnostic marker and therapeutic target for cancer patients and can predict poor prognosis.

To improve the prognosis of patients with cancer, an increasing number of studies have identified biomarkers that can predict cancer prognosis through bioinformatics analysis, such as Zhao et al. who found that aberrant expression of *STEAP1* in pancancer predicted survival and CFS and could be a potential therapeutic target [50]. Chen et al. suggested that *ALKBH7* may serve as a potential prognostic pancancer biomarker and is involved in the immune response [51]. Thus, we investigated the expression levels of MCM3AP-AS1 in cancers through the GEPIA database. The outcomes showed that MCM3AP-AS1 was overexpressed in various cancers, and patients in the high-expression group had poor OS. Then, we selected MCM3AP-AS1-related genes from the MEM databases, performed GO and KEGG enrichment analysis, and constructed a signaling network to better define the functions of MCM3AP-AS1 in cancers. The outcomes of GO analysis revealed that MCM3AP-AS1 has a lot to do with the nucleoplasm, nucleus, transcription, and poly(A) RNA binding. Furthermore, the results of KEGG analysis revealed that MCM3AP-AS1 was significantly correlated with RNA transport, ribosome biogenesis in eukaryotes, spliceosome, and regulating pluripotency of stem cell-related signaling pathways. Moreover, we have further investigated the mechanism of MCM3AP-AS1 in cancers. In CRC, the high expression of lncRNA MCM3AP-AS1 promotes cell metastasis and proliferation by regulating miR-193a-5p/SENP1 [37]. MCM3AP-AS1 is upregulated in HCC and enhances the growth of HCC by targeting the miR-194-5p/FOXA1 axis [38]. In PC, MCM3AP-AS1 accelerates migration and growth through modulating FOXK1 by sponging miR-138-5p [22]. To investigate the association between MCM3AP-AS1 and multiple cancers, we concluded MCM3AP-AS1 and its functional role and related target genes, as shown in Table 4.

Notwithstanding, there are some limitations to our study. First, the literature we included was all from China, so there may be selection bias in our outcomes. Second, there is no uniform cut-off value for the MCM3AP-AS1 expression level, and the survival data HR and 95% CI for some studies were extracted by Engauge Digitizer software and may contain statistical errors. Third, only one of the included studies demonstrated that downregulation of MCM3AP-AS1 was linked to the survival of CC.

## 5. Conclusions

In conclusion, our meta-analysis demonstrated that overexpression of MCM3AP-AS1 in cancers was significantly associated with poor survival and CFS. Furthermore, MCM3AP-AS1 can be considered a novel prognostic biomarker and therapeutic target for various cancers. Nonetheless, our study has some limitations, and these conclusions need to be confirmed by additional high-quality, large sample size, and multicenter studies.

## Figures and Tables

**Table 1:** Characteristics of the included studies.

Cancer	First author	Year	Country	Sample type	Sample size (*n*)	Detection method	Cut-off value	Outcome	Hazard ratios	NOS score
CC [35]	Lan L	2020	China	Tissue	64	qRT-PCR	Median	OS	K-M curve	8
CRC [16]	Dai WX	2021	China	Tissue	53	qRT-PCR	Median	CFS	NR	7
CRC [36]	Ma XD	2021	China	Tissue	60	qRT-PCR	Median	OS, CFS	K-M curve	8
CRC [37]	Zhou MY	2021	China	Tissue	100	qRT-PCR	NR	OS	K-M curve	7
EC [17]	Yu J	2021	China	Tissue	60	qRT-PCR	Median	OS, CFS	K-M curve	8
HCC [38]	Wang YF	2019	China	Tissue	80	qRT-PCR	Median	OS, CFS	K-M curve	8
LC [19]	Luo H	2021	China	Tissue	60	qRT-PCR	Median	OS	K-M curve	8
LC [39]	Shen DJ	2021	China	Tissue	63	qRT-PCR	NR	CFS	NR	6
Lymphoma [40]	Guo C	2020	China	Tissue	41	qRT-PCR	NR	OS, CFS	K-M curve	7
NPC [20]	Sun PY	2020	China	Tissue	55	qRT-PCR	Median	OS	K-M curve	8
OSCC [21]	Hou C	2020	China	Tissue	36	qRT-PCR	Median	CFS	NR	7
PC [22]	Yang M	2019	China	Tissue	86	qRT-PCR	Median	OS, CFS	K-M curve	8
PTC [41]	Liang MH	2019	China	Tissue	68	qRT-PCR	Median	OS	K-M curve	8
PCa [23]	Jia ZH	2020	China	Tissue	64	qRT-PCR	Mean	OS, CFS	K-M curve	8
PCa [42]	Li X	2020	China	Tissue	46	qRT-PCR	Mean	RFS	K-M curve	8
RCC [24]	Qiu L	2020	China	Tissue	78	qRT-PCR	Median	OS, RFS	K-M curve	8

CC: cervical carcinoma; CRC: colorectal cancer; EC: endometrioid carcinoma; HCC: hepatocellular carcinoma; LC: lung cancer; NPC: nasopharyngeal carcinoma; OSCC: oral squamous cell carcinoma; PC: pancreatic cancer; PTC: papillary thyroid cancer; PCa: prostate cancer; RCC: renal cell carcinoma; qRT-PCR: quantitative real-time polymerase chain reaction; NR: not reported; OS: overall survival; RFS: relapse-free survival; CFS: clinicopathological features.

**Table 2:** Association of MCM3AP-AS1 expression with clinicopathological features.

Clinicopathological parameters	Studies (*n*)	Patients (*n*)	OR (95% CI)	*P* value	Heterogeneity (*I*^2^, *P*)	Model
Age (elderly vs. young)	8	479	0.81 (0.56, 1.17)	0.263	0.0%, 0.894	Fixed
Gender (male vs. female)	7	419	0.98 (0.65, 1.46)	0.902	0.0%, 0.631	Fixed
Tumor size (large size vs. small size)	5	323	2.34 (0.84, 6.51)	0.105	78.4%, 0.001	Random
TNM stage (III + IV vs. I + II)	8	463	2.28 (1.14, 4.54)	0.019	64.4%, 0.006	Random
Lymph node metastasis (positive vs. negative)	5	299	2.97 (1.83, 4.83)	<0.001	43.6%, 0.131	Fixed
Differentiation grade (poor VS well/moderate)	4	260	2.65 (1.54, 4.58)	0.018	0.0%, 0.732	Fixed

**Table 3:** Gene ontology analysis of the lncRNA MCM3AP-AS1-related genes.

GO number	Description	Genes	*P* value
GO:0044822	Poly(A) RNA binding	TCERG1, SMG1, DDX42, CCAR1, TIAL1, PNN, RBM34, NOM1, PNISR, NSUN2, WDR75, ALG13, PRPF4B, GNL2, DNAJC2, SFPQ, DDX39A, SLTM, NFX1, PSPC1, HNRNPH1, SRSF3, LUC7L3, SREK1, HNRNPH3, SNRNP200, NSRP1, SRSF7, LUC7L2, RBM22, ZCCHC8, YTHDC1, SRRT, AKAP8, NOLC1, NOL8, AKAP17A, C1ORF52, EXOSC10, HNRNPDL, TRA2B, RBBP6, HNRNPA1, SMNDC1, SRSF11, NOP58, TIA1, HNRNPA3, PRPF38B, ZRANB2, CCDC59, PRRC2C, UPF3B, NAP1L1, NOC3L, U2SURP, SNW1, FUBP1, HNRNPA2B1, CEBPZ, RBMX	1.50*E*-32
GO:0005654	Nucleoplasm	MDC1, SETD2, ICE1, GORAB, DDX42, FAM208B, ARID4B, ZNF45, CCAR1, MED17, TIAL1, PNN, UIMC1, GPBP1, CCNL2, RIOK1, FNBP4, PNISR, USP48, KDM2A, ESCO1, NSUN2, WDR75, HAUS3, PRPF4B, THOC2, DNAJC2, SFPQ, DDX39A, DMTF1, SLTM, PSPC1, HNRNPH1, SRSF3, LUC7L3, ANAPC4, SREK1, HNRNPH3, BDP1, SNRNP200, SRSF7, RBM22, ATF4, ZCCHC8, KDM3A, UHRF2, RNMT, SRRT, AKAP8, NOLC1, AKAP17A, PDS5A, EIF1AD, TAF1D, EXOSC10, BTAF1, HNRNPDL, TRA2B, TGS1, UBR5, EXOSC8, RBBP6, HNRNPA1, ARGLU1, ZBED5, SRSF11, NOP58, TIA1, HNRNPA3, ZRANB2, CCDC59, TAF11, UPF3B, U2SURP, SNW1, KANSL2, FUBP1, HNRNPA2B1, ERCC5, MDM4, OGT, RBMX, PTPN2, EZH2	7.37*E*-31
GO:0005634	Nucleus	TCERG1, ZNF451, MDC1, GORAB, ARID4B, TIAL1, GPBP1, CCNL2, NEPRO, NOM1, KDM2A, USP3, IFRD1, SUPT7L, DDX39A, DMTF1, PSPC1, ANAPC4, FAM76B, SNRNP200, SRSF7, ATF4, ZCCHC8, YTHDC1, AKAP17A, ZNF23, C1ORF52, PDS5A, EIF1AD, HNRNPDL, UBR5, ARGLU1, MGEA5, ZRANB2, UPF3B, CLK4, CLK1, GON4L, SNW1, FUBP1, MDM4, RBAK, OGT, EZH2, CREBZF, SMG1, SETD2, DDX42, FAM208B, CHD1, MYSM1, N4BP2L2, MED17, DUSP12, UIMC1, RBM34, OFD1, USP47, ZNF121, NSUN2, GNL2, DNAJC2, SFPQ, LSG1, SLTM, HNRNPH1, NFX1, LUC7L3, HNRNPH3, NSRP1, RBM22, KDM3A, UHRF2, RNMT, SFSWAP, AKAP8, NOL8, EXOSC10, TRA2B, TGS1, EXOSC8, RBBP6, ZNF621, HNRNPA1, SMNDC1, SRSF11, HNRNPA3, CDK11A, NOP58, CCDC59, NAP1L1, NOC3L, U2SURP, HNRNPA2B1, ERCC5, CEBPZ, NAA16, PAXBP1, RBMX, PTPN2	2.97*E*-21
GO:0000166	Nucleotide binding	SRRT, NOL8, AKAP17A, TIAL1, EXOSC10, HNRNPDL, TRA2B, HNRNPA1, RBM34, SRSF11, RBM17, TIA1, HNRNPA3, UPF3B, U2SURP, SFPQ, SLTM, PSPC1, HNRNPH1, HNRNPA2B1, SRSF3, SREK1, HNRNPH3, SRSF7, RBMX, RBM22	2.69*E*-16
GO:0000398	mRNA splicing	ZCCHC8, HNRNPA3, SRRT, UPF3B, PRPF4B, CCAR1, PNN, DDX39A, SNW1, PSPC1, HNRNPH1, TRA2B, HNRNPA2B1, SRSF3, HNRNPH3, HNRNPA1, SNRNP200, SRSF7, RBMX, SRSF11, RBM22	2.69*E*-15
GO:0016607	Nuclear speck	PNISR, YTHDC1, DDX42, AKAP17A, THOC2, NOC3L, PNN, PSPC1, LUC7L3, SRSF3, FAM76B, CCNL2, NSRP1, SMNDC1, LUC7L2	9.22*E*-10
GO:0071013	Catalytic step 2 spliceosome	ZCCHC8, PNN, HNRNPA3, SNW1, HNRNPH1, HNRNPA2B1, PRPF4B, HNRNPA1, SNRNP200, RBMX, RBM22	3.51*E*-09
GO:0005681	Spliceosomal complex	RBM17, DDX39A, SNW1, HNRNPDL, HNRNPA2B1, AKAP17A, HNRNPH3, SREK1, HNRNPA1, SNRNP200, SMNDC1	4.34*E*-09
GO:0003676	Nucleic acid binding	DDX42, NOL8, ZNF23, ZNF45, TIAL1, HNRNPDL, TRA2B, RBBP6, ZNF621, HNRNPA1, RBM34, SRSF11, ZNF121, RBM17, TIA1, HNRNPA3, U2SURP, SFPQ, DDX39A, SLTM, PSPC1, HNRNPH1, HNRNPA2B1, SRSF3, SREK1, HNRNPH3, SNRNP200, RBAK, SRSF7, RBMX	5.09*E*-09
GO:0008380	RNA splicing	SFPQ, PRPF38B, ZRANB2, LUC7L3, PRPF4B, AKAP17A, THOC2, HNRNPH3, SREK1, NSRP1, SRSF7, SRSF11	1.54*E*-07

**Table 4:** Summary of lncRNA MCM3AP-AS1 functional roles and related genes.

Cancer type	Expression	Function roles	Related genes	Reference
Cervical cancer	Downregulate	Cell proliferation	*miR-93*	[35]
Colorectal cancer	Upregulate/downregulate	Cell proliferation, migration, cycle, colony formation, and invasion	*miR-545*, *CDK4*, *miR-19a-3p/FOXF2*, *miR-193a-5p/SENP1*	[16, 36, 37]
Endometrioid carcinoma	Upregulate	Cell invasion and migration	*miR-126/VEGF*	[17]
Hepatocellular carcinoma	Upregulate	Cell proliferation, colony formation, cycle progression, and apoptosis	*miR-194-5p/FOXA1*	[38]
Lung cancer	Upregulate	Cell proliferation, migration, and invasion	*miR-148a, miR-195-5p*	[19, 39]
Lymphoma	Upregulate	Cell growth and apoptosis	*miR-15a/EIF4E*	[40]
Nasopharyngeal carcinoma	Upregulate	Cell proliferation and apoptosis	*miR-34a*	[20]
Oral squamous cell carcinoma	Upregulate	Cell proliferation, migration, and invasion	*miR-363-5p*	[21]
Pancreatic cancer	Upregulate	Cell proliferation, migration, and invasion	*miR-138-5p/FOXK1*	[22]
Papillary thyroid cancer	Upregulate	Cell proliferation, migration, and invasion	*miR-211-5p/SPARC*	[41]
Prostate cancer	Upregulate	Cell proliferation, migration, and invasion	*miR-543-3p/SLC39A10/PTEN*, *DNMT1/DNMT3*, *NPY1R*	[23, 42]
Renal cell carcinoma	Upregulate	Cell proliferation	*DPP4*	[24]

## Data Availability

The datasets used and/or analyzed during the current study are available from the corresponding author on reasonable request.
